# From Plexiglas to hologram: a path for layered artworks

**DOI:** 10.1098/rsos.250874

**Published:** 2025-09-11

**Authors:** Philippe Gentet, Yosman Botero Gomez, Seung-Hyun Lee

**Affiliations:** ^1^ Department of Immersive Convergence Content, Kwangwoon University, Nowon-gu, Republic of Korea; ^2^ University of Barcelona, Barcelona, Spain

**Keywords:** holostereosynthesis, multilayer painting, digital holography, holographic imaging, art and technology integration

## Abstract

We demonstrate the full-parallax holographic reproduction of a multilayer acrylic painting using the CHIMERA holographic printing system. The original artwork, composed of hand-painted transparent layers, was digitized, spatially reconstructed via holostereosynthesis in a three-dimensional environment and printed on a silver halide plate. This approach preserves spatial fidelity and artistic intent, offering a bridge between fine art and holography, with potential applications in cultural preservation, immersive displays and art education.

## 1. Introduction

The representation of three-dimensionality in visual art has long been a central concern regarding aesthetic and technical aspects [1–3]. While sculptures and physical installations naturally occupy space, contemporary artists are increasingly exploring hybrid methods that combine painting, transparency and spatial layering to create depth illusions from two-dimensional formats. One such innovative approach involves the superimposition of hand-painted or printed elements on a transparent substrate, such as glass, acrylic or polyvinyl chloride, to construct spatially represented artwork [4].

Colombian artist Yosman Botero, also known as Yoshbott, stands out for his systematic use of multilayer techniques [5]. By distributing the elements of composition across several transparent panels—each individually painted and then assembled in depth—he creates pieces that may be described as sculpted paintings or relief images. This approach results in a dynamic and immersive viewing experience in which the image varies depending on the observer’s perspective.

Other artists have explored similar methodologies. David Spriggs constructs volumetric installations using hand-painted transparent sheets to produce ephemeral spatial forms that appear suspended in mid-air [6]. Nobuhiro Nakanishi’s layer drawings involve printed photographic sequences on acrylic sheets aligned in parallel to evoke a three-dimensional temporal landscape [7]. Xiaowan Xia, a Chinese artist, creates intricate portraits and compositions using multiple layers of acrylic glass, paints them with subtle variations and assembles them to produce delicate volumetric illusions, blurring the line between painting and sculpture [8]. These works reflect an interest in stratified visual experiences situated at the crossroads of painting, sculpture and optics.

**Figure 1.**
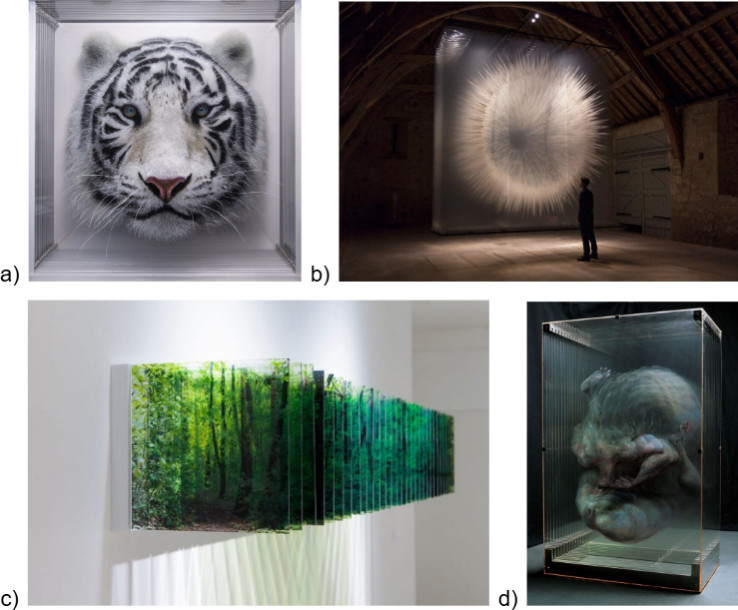
Examples of multilayered artwork by contemporary artists. (a) *Taxonomy 110* (2023) by Yosman Botero [5], composed of hand-painted transparent layers assembled in depth. Acrylic on Plexiglas of 52 × 52 × 21 cm. (b) Installation (2017) by British–Canadian artist David Spriggs [6], composed of hand-painted transparent sheets with dimensions of 5 × 2 × 5 m. (c) Three-dimensional landscapes by Nobuhiro Nakanishi [7], created using sequential photographic prints on acrylic sheets (© Nobuhiro Nakanishi, Courtesy of Yumiko Chiba Associates). (d) A 2002 work by Xiaowan Xia [8], constructed from multiple painted layers of acrylic glass.

Representative examples of these artists’ works are shown in figure 1, which illustrates the diversity and conceptual convergence of multilayered practices in contemporary visual art.

The convergence of visual art and optics echoes the long tradition of Renaissance artists working on the frontier of technological discovery, as discussed by Hockney [9]. Similarly, contemporary artists engage with emerging imaging technologies, such as virtual reality or artificial intelligence, to reimagine perception. This return to optics, which blends traditional techniques with digital innovation, opens new paths for visual art to evolve in tandem with scientific and technological tools. In this context, multilayered transparent artwork offers a compelling medium for exploring the dimensionality, perception and boundaries between its visible and hidden aspects. However, despite the artistic interest in multilayer compositions, there is a lack of research focused on converting such spatial artworks into high-fidelity digital holograms.

We examine the potential of multilayer techniques from an artistic viewpoint in the context of digital reproduction and holographic visualization. By integrating artwork into a holostereosynthesis workflow first described by Gentet *et al.* [10] and using the CHIMERA holographic printing system (holoprinter for short) [11], we investigated the faithful translation of physical multilayer composition into a full-parallax hologram without compromising the spatial fidelity and artistic intent [12–16].

## 2. Material and methods

**Figure 2.**
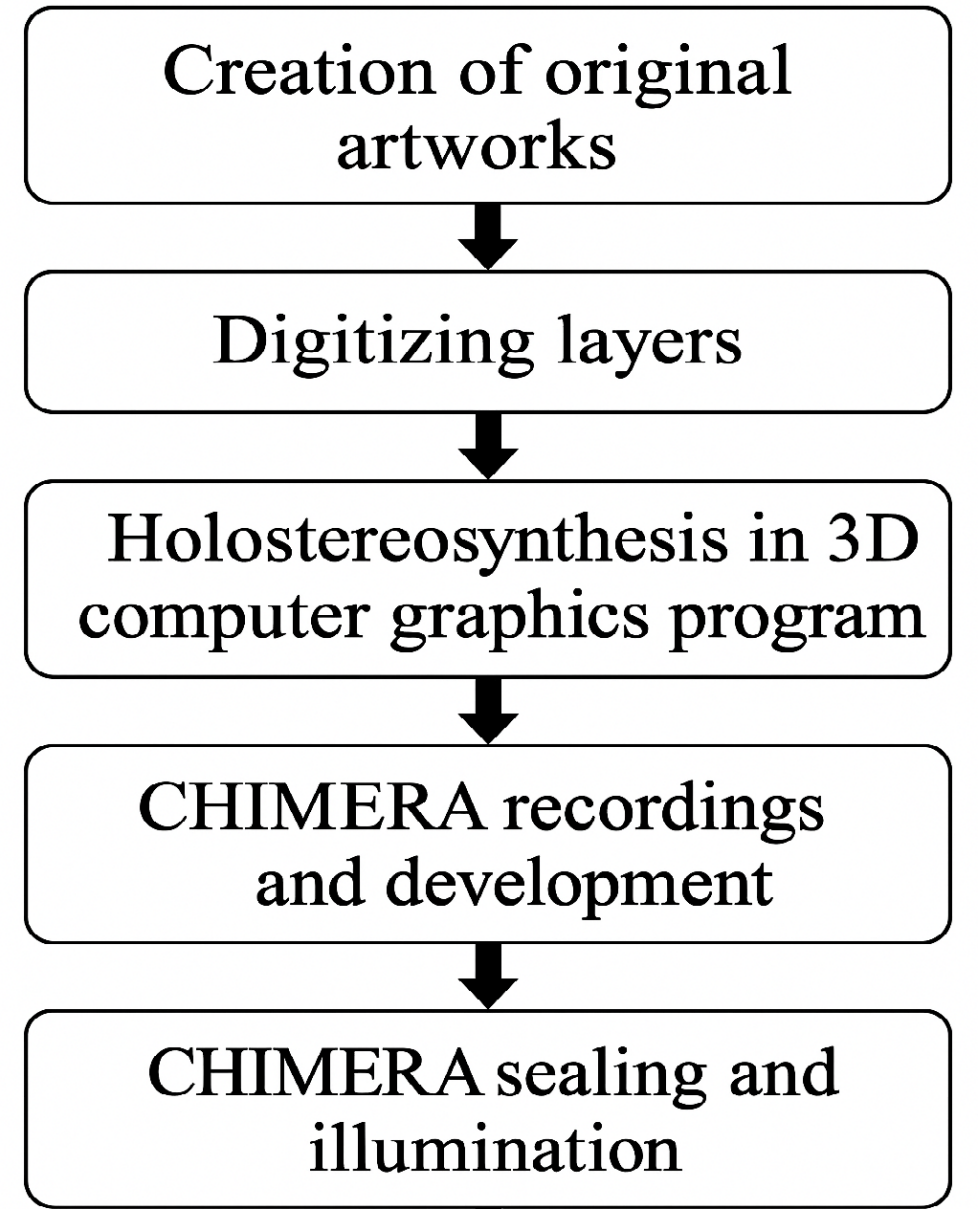
Study workflow.

This section presents the study workflow from the original artwork to the final hologram. Figure 2 shows an overview of the adopted process, and each step is detailed in the following subsections.

### 2.1. Creation of original artworks

Each artwork layer can be created using a range of techniques, either hand-painted creations with materials such as pastel, acrylic and oil directly onto transparent substrates including glass, acrylic sheets and polyvinyl chloride, or digital illustrations using graphic software and then printed onto the same transparent supports using high-resolution printers. This flexibility allows for a blend of traditional and digital methods, adapting to the artist’s preferred workflow and desired aesthetics of the final piece. The number of layers depends on the complexity of the image and the intended size of the final artwork.

### 2.2. Digitizing layers

Scanning hand-painted layers presents several challenges. White or lightly coloured paint details often become nearly invisible against a white background, making it difficult to capture the full intricacy of artwork containing such details. In addition, strong lighting used during scanning may create unwanted shadows or glare, particularly when textured or reflective surfaces are scanned. To overcome these issues, a soft grey background during photography or scanning allows for enhancing the contrast and improving the visibility of subtle details, resulting in higher-quality digital reproductions of each layer. On the other hand, layers created digitally using graphic software are inherently optimized for use and do not require scanning or streamlining of the workflow.

Once digitized or created, all layers are composited onto a black background using a raster graphics editor (e.g. Photoshop and GIMP) to facilitate accurate holostereosynthesis during holographic reconstruction.

### 2.3. Holostereosynthesis in a three-dimensional computer graphics program

Holostereosynthesis is the modern adaptation of Louis Lumière’s early twentieth-century photostereosynthesis, in which multiple depth planes or layers are digitally stacked and optically fused into a single coherent volumetric image using holographic printing. To perform holostereosynthesis using the CHIMERA holoprinter, each scanned layer of artwork was imported into a three-dimensional computer graphics environment (Blender, 3ds Max or Maya) and arranged symmetrically around a central rotation axis, with half of the layers placed in front and half behind. The spatial positioning of the layers was preserved according to their original depth in the artwork and scaled proportionally based on the chosen scale within the three-dimensional scene.

A cylindrical virtual camera rig was used to generate a matrix of 121 × 121 perspective images per layer, covering a 60° viewing arc. This was achieved by rotating a virtual camera around the scene in each layer. Once all the layers were rendered, the resulting image sets were combined using custom in-house software to produce a unified 121 × 121 perspective dataset.

### 2.4. CHIMERA recordings and development

Custom in-house software was used to process the set of perspective images and generate the corresponding hogel data [17] required for holographic printing. Each hogel was sequentially recorded using a high-precision RGB (red–green–blue) display system composed of three spatial light modulators and one 120° full-colour optical printing head. The holographic information per RGB hogel was encoded onto an Ultimate U04 silver halide glass plate [18] via interference with a coherent reference beam.

Each hogel measured 250 µm, and the system operated at a printing frequency of 60 Hz. The CHIMERA holoprinter used three 20  mW diode-pumped solid-state lasers at wavelengths of 640 (red), 532 (green) and 457  nm (blue). The Ultimate U04 plates were specifically designed for full-colour holography, being isopanchromatic to offer uniform sensitivity across the visible spectrum and featuring low light-scattering properties to ensure high image clarity and colour fidelity. The holograms were developed using two safe and user-friendly chemical baths and optimized for consistent results.

### 2.5. Hologram sealing and illumination

To ensure accurate reconstruction, the final hologram must be illuminated with the same wavelengths used during recording. To maintain long-term dimensional stability and prevent any deformation of the emulsion (e.g. swelling and shrinkage) owing to fluctuations in humidity or temperature, a protective glass plate was bonded to the hologram using optical-grade ultraviolet-curable glue.

Reconstruction requires a point light source that includes the original recording wavelength. RGB light-emitting diodes (LEDs) are suitable for this purpose because their emission peaks closely match the wavelengths of the recording lasers [19]. For optimal display, an RGB LED was positioned 50 cm from the centre of the hologram at 45° to provide uniform and adequate illumination.

## 3. Results

### 3.1. Original artwork

**Figure 3.**
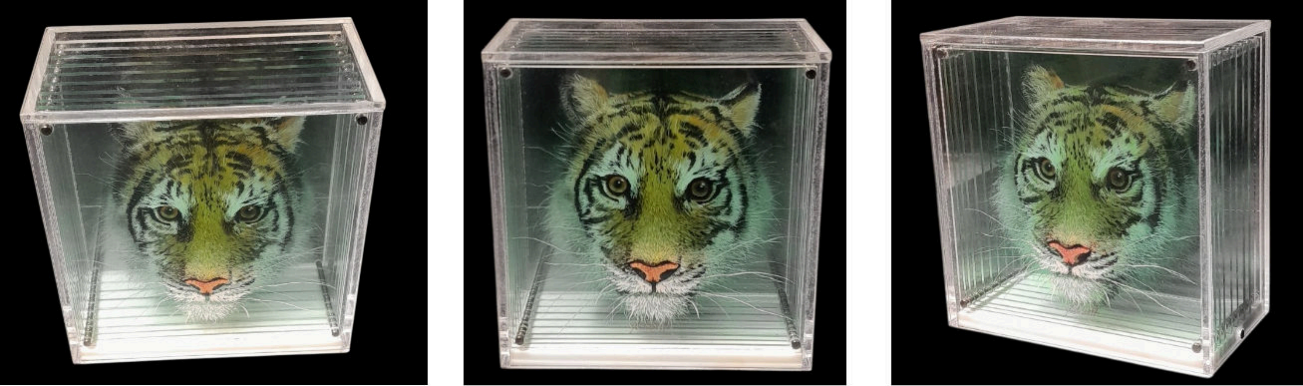
*Taxonomy Test 1* by Yosman Botero [5]. Acrylic on Plexiglas with dimensions of 17 × 16 × 9 cm. Top, front and side views of an original multilayered artwork.

The artwork featured in this holostereosynthesis experiment was *Taxonomy Test 1*, a 2024 creation by artist Yosman Botero, shown in figure 3. This piece depicts a tiger head (a symbol of Korea), and it is painted in acrylic on transparent Plexiglas in nine hand-painted layers, each contributing to a complex spatial composition. The physical dimensions of the study area are 17 × 16 × 9 cm. The layered arrangement and choice of materials make it particularly suitable for holographic reconstruction, offering both depth and visual richness that are ideal for the CHIMERA holoprinter.

The transparency of the support allows the layers to remain visibly distinct when viewed from oblique angles, whereas the accumulated thickness of the acrylic substrate introduces slight optical diffusion and localized blurring.

### 3.2. Digitizing layers

**Figure 4.**
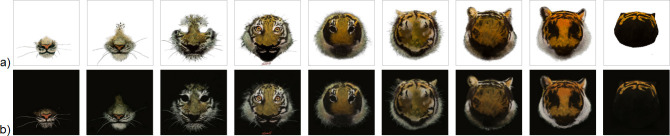
Digitization and preprocessing of original hand-painted layers. (a) Scanned acrylic layers photographed against a grey background for contrast enhancement. (b) Corresponding layers composited onto a black background for holostereosynthesis.

The nine individual layers were digitized using a high-resolution digital camera (Canon 5D Mark III) and photographed against a soft grey background to enhance the contrast and improve the visibility of subtle painted details. This approach helped minimize reflections and preserve the tonal variation across transparent surfaces, resulting in a high-quality digital reproduction (figure 4a). Once digitized, the layers were composited onto a uniform black background (figure 4b) using Adobe Photoshop. This step was essential for preparing files for accurate holostereosynthesis, ensuring clean separation between the image content and transparent regions during holographic reconstruction.

### 3.3. Holostereosynthesis

**Figure 5.**
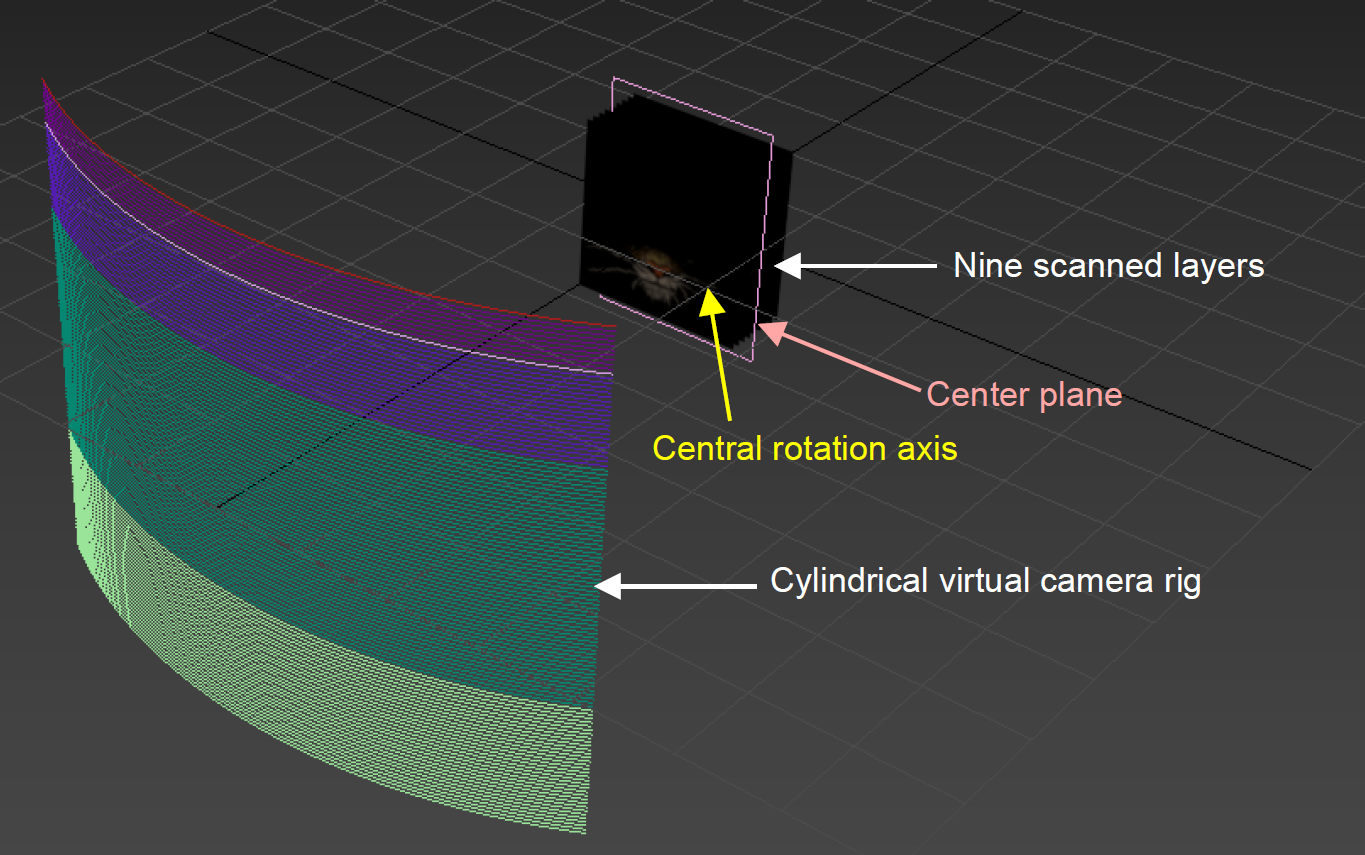
Virtual three-dimensional arrangement of nine scanned layers in Autodesk 3ds Max for holostereosynthesis. Four layers were positioned in front and four behind the central rotational axis, thereby preserving the spatial depth of the original artwork.

To perform holostereosynthesis, the nine scanned layers were imported into Autodesk 3ds Max and arranged symmetrically around a central rotational axis. Four layers were positioned in front of the centre plane and four were positioned behind it, maintaining the relative depth relationships of the original physical artwork (figure 5). The spatial configuration of each layer was preserved and scaled proportionally according to the overall dimensions of the original piece and scale defined within a virtual three-dimensional environment.

**Figure 6.**
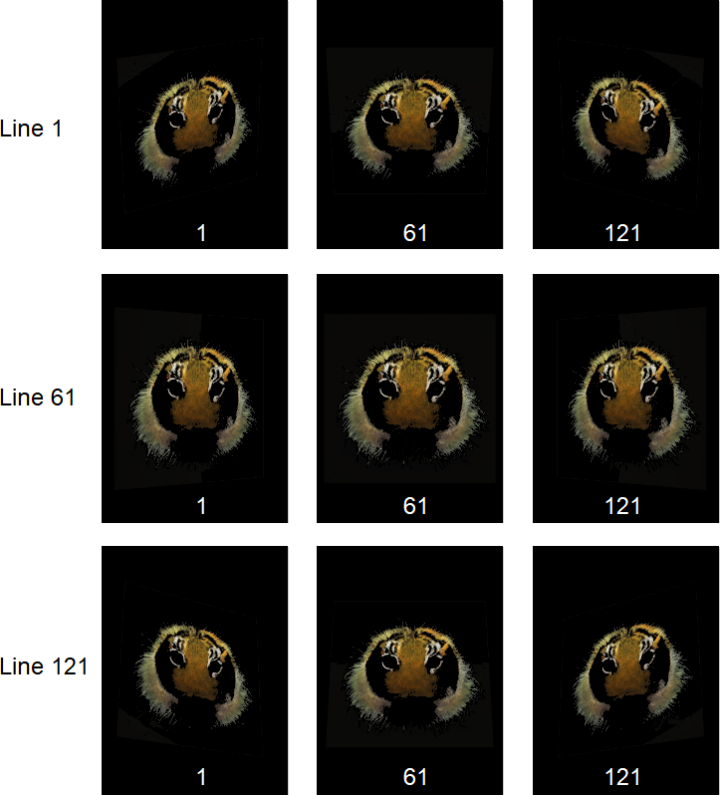
Selected perspective images of the central layer were generated using a cylindrical virtual camera rig over a 60° viewing arc.

A cylindrical virtual camera rig was used to generate a matrix of 121 × 121 perspective images per layer, covering a 60° viewing arc. This result was achieved by rotating the virtual camera around the scene and capturing views at evenly spaced angular intervals of 0.5°. Each image was rendered at a resolution of 1320 × 1760 pixels to ensure high-fidelity details for stereoscopic depth reconstruction. Figure 6 shows a selection of perspective images obtained for the central layer of the composition. This dense angular sampling was crucial for accurate light-field synthesis and high-quality visualization.

**Figure 7.**
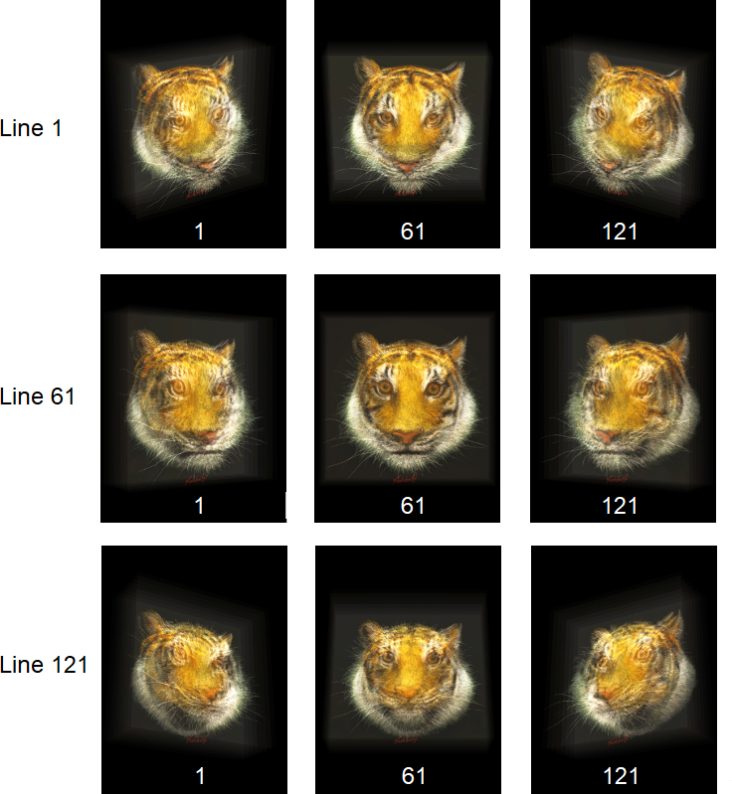
Sample perspective images from a fully fused multilayer composition, illustrating combined depth and spatial coherence of the nine holostereosynthesized layers.

Once all layers were rendered, the resulting sets of perspective images were merged using custom in-house software to produce a unified 121 × 121 perspective dataset representing the complete volumetric composition. Figure 7 shows a selection of perspective views generated from a fused multilayer scene.

### 3.4. Final hologram

**Figure 8.**
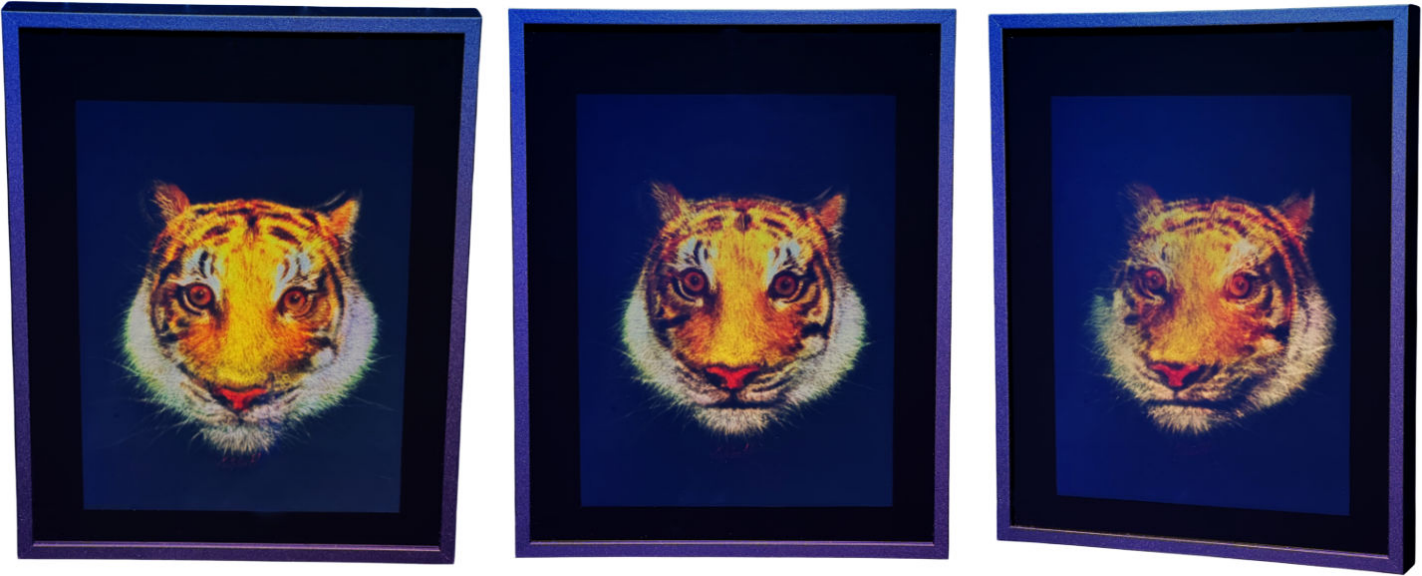
Top, front and side views of full-colour hologram of *Taxonomy Test 1* reconstructed in CHIMERA from nine holostereosynthesized layers. The hologram was printed on 15 × 20 cm U04 plates under illumination of an RGB LED.

The corresponding hogels were generated from the computed perspective images and sequentially recorded on a 15 × 20 cm U04 holographic plate using the CHIMERA holoprinter. Under illumination of an RGB LED source, the hologram produced a vivid full-colour three-dimensional reconstruction of the tiger head, as shown in figure 8 and electronic supplementary material, video S1. The holostereosynthesis integration of the original nine layers was achieved through a single hologram exposure, demonstrating the capacity of the CHIMERA holoprinter to condense multilayered spatial data into a unified record.

The resulting three-dimensional image appears luminous and exhibits strong depth cues, with the illusion of protrusion beyond the physical boundaries of the plate. Colour rendering is notably more vibrant than that in the original artwork, with the enhanced saturation and brightness contributing to a heightened visual impact. Owing to the 250 µm hogel resolution, the hogel grid is imperceptible to the naked eye, ensuring visual continuity across the entire image. The hologram offers a full-parallax field of view extending 60° along both the horizontal and vertical axes, enabling immersive three-dimensional perception from multiple viewing angles. Notably, this hologram possesses mastering-grade quality and is suitable for replication. Using the same RGB lasers, the hologram can be duplicated on silver halide or photopolymer media without perceptible degradation of the resolution or colour fidelity.

Compared with the original artwork, several key differences emerge. First, the background of the holographic reconstruction is black rather than the original transparent. Second, the image appears to float in space and is no longer confined to a Plexiglas cube, thereby enhancing the sensation of spatial freedom. Third, the field of view is intentionally limited to 60° horizontally and vertically to suppress the visibility of the discrete layered structure and avoid revealing the individual depth planes. More importantly, the layers appear seamlessly fused, contributing to a coherent and realistic impression of volumetric depth in the three-dimensional image. These features collectively produce a new artistic object that emerges as a distinct and transformative reinterpretation while retaining the essence of the original artwork.

## 4. Discussion

The results of this study demonstrate the feasibility of holostereosynthesis to transform multilayer acrylic artworks into full-parallax full-colour holograms without compromising their spatial or aesthetic fidelity. The CHIMERA holoprinter translates complex spatial compositions of hand-painted transparent layers into volumetric light fields, producing holographic images that preserve both the depth cues and visual richness of the original artwork.

A key insight from this study is the compatibility between the multilayer painting technique (used by Yosman Botero and other artists) and holostereosynthesis. Once digitized and loaded in a three-dimensional graphics environment, each layer retains its visual integrity when rendered into perspective image matrices. The preservation of the relative spatial positioning, contrast and painted details is critical for maintaining the immersive experience of holographic reconstruction.

The use of custom in-house software and a cylindrical virtual camera rig enables efficient and precise perspective capture, ensuring the smooth integration of layers into a single coherent holographic dataset. This approach supports high-resolution encoding across a wide viewing angle (60 × 60 perspectives over 60°), which is a key requirement for producing high-quality holograms that are suitable for public exhibitions.

The U04 plates further contribute to the visual fidelity of the hologram. Their isopanchromatic response and low light-scattering properties ensure accurate colour reproduction and minimal background noise. Additionally, sealing the plates under a second glass layer protects them from environmental variations, thereby preserving their long-term optical performance.

A notable advantage of this method is its flexibility. While traditional holography often requires live subjects or object scans, holostereosynthesis is uniquely suited to artworks that are already inherently spatial. This opens a path to wider applications among contemporary artists who work with transparent layered media, offering them a means to create reproducible and scalable holographic versions of their original compositions.

This study also highlights the importance of careful layer digitization. While digitally created layers integrate seamlessly into the process, hand-painted layers require careful scanning. The application of a soft grey background and controlled lighting is essential for retaining subtle details, particularly in white or pastel-painted areas. These scanning conditions ensure that no artistic nuance is lost in the translation from the physical medium to light-based reproduction.

The resulting hologram from CHIMERA can provide an engaging three-dimensional experience to viewers, matching the original spatial layering and enabling dynamic perception from multiple angles. In some aspects, the holographic version can enhance the viewer’s experience through improved colour saturation, perceived luminosity and the illusion of the artwork floating freely in space. These capabilities suggest a strong potential for future use in museums, galleries and installations to showcase depth-rich artworks while avoiding physical space limitations and potential artwork damage.

## 5. Conclusion

We demonstrate that multilayer transparent artwork can be translated into full-parallax full-colour holograms using holostereosynthesis and a CHIMERA holoprinter. The developed process preserves the spatial and aesthetic qualities of the original artwork. In addition to artistic preservation, our proposal seems promising for exhibitions in museums, galleries and educational settings, possibly enabling broad access to three-dimensional artwork through portable immersive holographic displays.

## Data Availability

Electronic supplementary material available at Figshare [20].
